# RETRACTION: Effect of Body Mass Index on the Wound Infection and Complications in Patients With Liver Cancer: A Meta‐Analysis

**DOI:** 10.1111/iwj.70444

**Published:** 2025-03-26

## Abstract

**RETRACTION:** LiY.‐P. , GanZ. , and ShiB. , “Effect of Body Mass Index on the Wound Infection and Complications in Patients With Liver Cancer: A Meta‐Analysis,” International Wound Journal 21, no. 2 (2024): e14689, 10.1111/iwj.14689.PMC1082756742052914

The above article, published online on 30 January 2024, in Wiley Online Library (http://onlinelibrary.wiley.com/), has been retracted by agreement between the journal Editor in Chief, Professor Keith Harding; and John Wiley & Sons Ltd. Following an investigation by the publisher, all parties have concluded that this article was accepted solely on the basis of a compromised peer review process. The editors have therefore decided to retract the article. The authors did not respond to our notice regarding the retraction.

**RETRACTION:** Y.‐P. Li , Z. Gan , and B. Shi , “Effect of Body Mass Index on the Wound Infection and Complications in Patients With Liver Cancer: A Meta‐Analysis,” International Wound Journal 21, no. 2 (2024): e14689, 10.1111/iwj.14689.PMC1082756742052914

The above article, published online on 30 January 2024, in Wiley Online Library (http://onlinelibrary.wiley.com/), has been retracted by agreement between the journal Editor in Chief, Professor Keith Harding; and John Wiley & Sons Ltd. Following an investigation by the publisher, all parties have concluded that this article was accepted solely on the basis of a compromised peer review process. The editors have therefore decided to retract the article. The authors did not respond to our notice regarding the retraction.

